# Inpatient rehabilitation fracture liaison service (FLS) improves outcomes for secondary prevention of hip fractures

**DOI:** 10.1016/j.bonr.2025.101869

**Published:** 2025-08-08

**Authors:** Orit Mazza, Chemda Gluck, Noa Menkes-Caspi, Robyn Jacob Bornstein, Hagay Amir, Michael Bahar, Amir Haim

**Affiliations:** a Loewenstein Rehabilitation Medical Center, Raanana, Israel; b Gray Faculty of Medical & Health Sciences Tel Aviv University, Tel Aviv, Israel; c Mental Health and Rehabilitation Research Center, Clalit Health Services, Petah-Tikva, Israel; d Clalit Health Services, Dan Petah-Tiqwa District, Israel; e Rabin Medical Center Beilinson Hospital, Petah Tikva, Israel

**Keywords:** Hip fractures, Fracture liaison service, Osteoporosis, Rehabilitation

## Abstract

**Background:**

Secondary fracture prevention is a well-defined treatment-gap within osteoporosis management. Fracture Liaison Service (FLS) coordinates the management and treatment of patients following a fragility fracture in order to close the care gap. We aim to assess the efficacy of the FLS initiative in the management and treatment of patients following fragility hip fracture in the inpatient rehabilitation setting.

**Methods:**

This is a diagnostic, retrospective cohort study using a deidentified, electronic health record database. In the extraction process, patients with fragility hip fractures were identified. Patients after major trauma or malignancy were excluded. The prevalence of initiation and adherence to anti-osteoporotic treatments, including alendronate, risedronate, zoledronate, denosumab, romosozumab, and teriparatide, was compared between the rehabilitation FLS initiative patients and patients from other hospitals without FLS.

**Results:**

A total of 4,124 patients with fragility hip fractures were identified between 2017 and 2021. The FLS initiative showed significantly higher rates of treatment initiation, with 72.1 % of patients receiving pharmacological therapy following a hip fracture, compared to 45.1 % in hospitals without FLS (p < 0.001). Patients in the FLS group also demonstrated higher rates of good adherence and lower rates of poor adherence (p < 0.001). Denosumab was the most commonly prescribed anti-osteoporotic treatment within the FLS initiative.

**Conclusions:**

The FLS in the inpatient rehabilitation setting was found to be highly effective in improving time to treatment initiation and adherence rates to prescribe anti-osteoporosis therapy. These findings demonstrate the role of FLS in addressing the osteoporosis treatment gap following fragility hip fracture.

## 1 Introduction

Osteoporosis is a growing public health concern, affecting hundreds of millions of people worldwide and impacting the quality and quantity of life (Camacho et al., 2020). The 2021 Scorecard for Osteoporosis in Europe (EU) documented the impact of osteoporosis in the European Union plus Switzerland and the United Kingdom (EU 27 + 2) in 2019 (Kanis et al., n.d.). According to World Health Organization (WHO) criteria, an estimated 22.5 million women and 6.5 million men have osteoporosis. The economic burden was estimated at € 57 billion, accounting for approximately 3.5 % of healthcare spending (Kanis et al., n.d.; Curtis et al., 2022; Willers et al., n.d.). With the ageing demographic, the projected rate of osteoporosis-related fractures is estimated to increase by 25 % by 2034 (Willers et al., n.d.).

The predominant clinical consequence of osteoporosis is bone fractures. Globally, an estimated one in three women and one in five men over the age of 50 will experience an osteoporotic fracture (Willers et al., n.d.). The most serious fracture types are hip and spine fractures, which are associated with considerable pain and disability (Willers et al., n.d.). Hip fracture is the most common reason for emergency anesthesia and surgery among older people and is the leading cause of death following a fall. Hip fractures are associated with reduced relative survival, with reported mortality rates of 5 % within one month and 26 % within 12 months of the fracture (Willers et al., n.d.; Gregson et al., 2022). Accordingly, secondary prevention following hip fracture has become a priority for healthcare systems worldwide (Gregson et al., 2022; Lems et al., 2017; Camacho et al., 2020).

There is a well-defined treatment gap for secondary prevention. 71 % (32–87 %) of women categorized as high-facture risk do not receive therapy which is considerably worse than the 2010 average of 55 % (Curtis et al., 2022). Previous studies confirm the potentially declining treatment rates (Curtis et al., 2022; Greenspan et al., 2012; Svedbom et al., 2013). The GLOW study of over 60,000 older women in 10 countries across the United States (US), the EU, and Australia reported that 80 % of women with a fragility fracture did not receive the recommended antiosteoporosis treatment (Curtis et al., 2022; Greenspan et al., 2012).

Several interventions have been suggested to address the secondary fracture prevention treatment-gap, including models based on screening, follow-up, and education for patients and clinicians (Kanis et al., n.d.; Lems et al., 2017). However, the gold standard model of care (MoC) for secondary prevention of osteoporosis is a FLS (Kanis et al., n.d.; Lems et al., 2017; Ganda et al., 2013; Wu et al., 2018). The FLS is a specialized, multidisciplinary service that coordinates the treatment process for patients following a fragility fracture. The core objectives of an FLS are (Camacho et al., 2020): identification (Kanis et al., n.d.) fracture risk, and clinical assessment (Curtis et al., 2022), intervention targeting secondary prevention (Willers et al., n.d.), and follow-up that maintains continuity of care (Eisman et al., 2012).

Most patients who experience a hip fracture undergo initial treatment in acute care and are then referred to a rehabilitation centre or program before returning to the community setting. The sub-acute phase of rehabilitation offers an optimal opportunity to effectively address the care gap. For this reason, in August 2017, the Rehabilitation Medical Center in our country, in conjunction with the Foundation for Osteoporosis and Bone Diseases, initiated an FLS centre in the inpatient rehabilitation setting.

To the best of our knowledge, the utility of the FLS program designed for rehabilitation has not been described. We have developed a program to address the treatment gap, specifically to provide comprehensive care for all patients presenting to our medical center with a fracture. The process included identification of patients, work-up, pharmacological and multidisciplinary treatment initiation, patient education, monitoring and management, treatment optimization, and follow-up. The primary objectives were to assess the effectiveness of this program as compared to the general population of patients who were treated in alternate local hospitals without FLS. We hypothesized that the FLS initiative would improve rates of time to treatment and adherence to prescribed anti-osteoporosis treatments.

## 2 Methods

This is a retrospective diagnostic cohort study using Clalit Health Services' automated electronic medical record database. The study was approved by the Institutional Ethics Committee for Research at the Levinstein Rehabilitation Medical Center (No.LOE-0004-22). All methods were performed in accordance with the relevant guidelines in the Helsinki Declaration. No informed consent was required.

### 2.1 Database

The variables extracted included demographic data: age, gender, and socioeconomic status (SES). SES was categorized as either low, intermediate, or high using geocoding techniques, where the address of the primary care clinic was linked to the census area-level SES data. The SES data were determined based on the rating provided by Israel's Central Bureau of Statistics (Filc et al., 2014). Data was collated regarding osteoporosis diagnosis and anti-osteoporosis medications (AOM) which included alendronate, risedronate, zoledronate, denosumab, romosozumab, and teriparatide. AOM selection was guided by current American Association of Clinical Endocrinologists/American College of Endocrinology (AACE/ACE) recommendations of the pharmacological treatment options for secondary prevention of osteoporotic fracture in the setting of the FLS clinic (Camacho et al., 2020). Current guidelines recommend the use of alendronate, risedronate, and zoledronate (bisphosphonates) as efficacious and safe for the prevention of hip fractures. Alternative options for patients with oral intolerance to bisphosphonates, and/or low compliance, or as initial treatment include abaloparatide, denosumab, romosozumab, and teriparatide (Grade A recommendation) (Camacho et al., 2020). In our research, the querying process included the treatments mentioned above.

### 2.2 Cohort selection

Adult patients aged between 50 and 85 years who experienced a fragility hip fracture between 2017 and 2021 were selected from the database. The *International Classification of Diseases, Tenth Revision* (*ICD-10*) codes were identified for fragility hip fracture as those resulting from minimal trauma or a fall from a standing height or less. Similar to other studies, the following codes were included: femoral neck fracture (S720), pertrochanteric fracture (code S721), and subtrochanteric fracture (code S722) (Quevedo et al., 2020; Yoon et al., 2013). The procedural codes for hip fracture surgical treatment during hospitalization were added to increase the accuracy of the hip fracture cohort. Patients with pathological fractures secondary to malignancy were excluded. Also, patients with major trauma after accidents (motor vehicle accidents, either as a driver, passenger, or pedestrian) were excluded to ensure identification of a potential fragility fracture resulting from osteoporosis. Patients were included in the FLS cohort if they were admitted to a rehabilitation facility with an FLS during the study period. Those admitted to a hospital without an FLS were included in the non-FLS cohort. No patient was included in both cohorts, and there was no overlap between the groups.

**Fig. 1:**
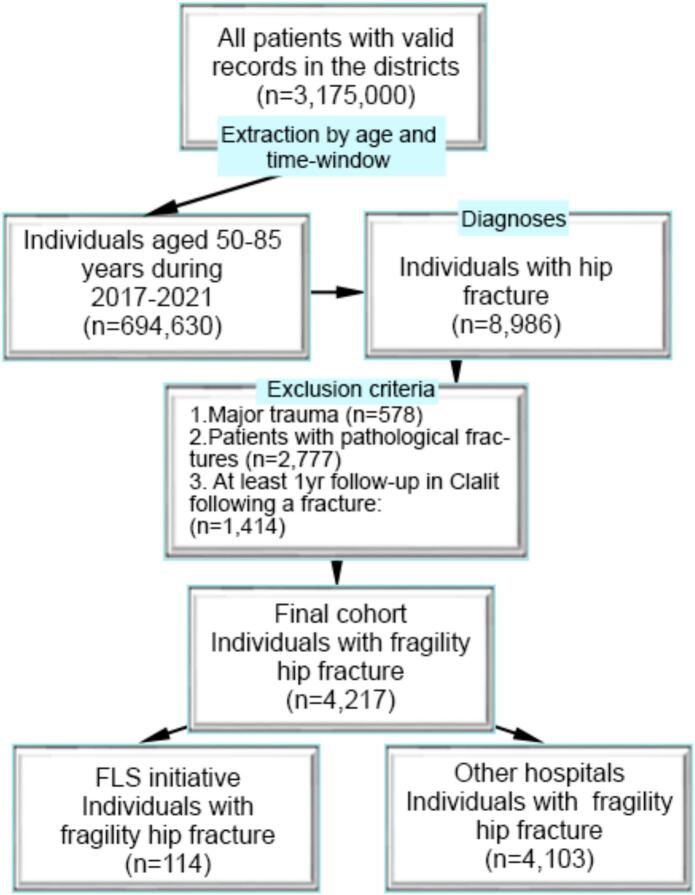
Cohort flowchart.

Fig. 1 demonstrates the cohort flowchart and data selection process. There were 3,175,000 patients within this catchment area with valid records. The first data extraction was conducted according to patient age and timeframe between January 2017 and June 2021, which resulted in 694,630 patients. Individuals with hip fractures were retrieved according to ICD-10 codes, which narrowed down the cohort to 8986 patients. Exclusion criteria were then applied, including history of major trauma, pathological fractures, and patients with less than 12 months of follow-up history within the database from the time of fracture (index date). The final cohort of individuals with fragility hip fractures consisted of 4217 patients. Within this cohort, 114 patients were admitted for rehabilitation during this time period and included in the FLS initiative. 111 of those patients were naïve to anti-osteoporosis medication. 4,103 patients with fragility hip fractures were admitted to other hospitals that didn't offer a FLS during this time period, 4013 of whom were naïve medication users. In all participating hospitals without an FLS program, rheumatology or endocrinology services were available; however, without a dedicated FLS, referrals and osteoporosis management were not systematic and largely depended on individual clinician decisions.

### 2.3 Primary and secondary outcomes

The primary outcome of the current study is the time to initiation of pharmacological treatment. This continuous dependent variable was calculated according to the difference between the index date of the fragility fracture and the first purchase date of prescribed AOM.

The International Osteoporosis Foundation (IOF) Capture the Fracture working group, with the Fragility Fracture Network (FFN) and National Osteoporosis Foundation (NOF), published a position paper in which they recommended eleven patient-level key performance indicators (KPIs) for FLS to guide quality improvement. According to their recommendations, FLS clinics should aim for initiation of AOM for secondary prevention within 12–16 weeks (Javaid et al., 2020). However, the US National Committee for Quality Assurance set a national measure for investigating and/or initiating treatment within 6 months (Javaid et al., 2020). Therefore, in this study, the evolution of osteoporosis management initiation was evaluated as a categorical variable, comprising three levels: good, good-moderate, and late. Good practice was defined by the initiation of those previously mentioned treatments within 3 months. Good-moderate was defined as initiation of treatment within 3–6 months from the index date. The late variable was defined as initiation of treatment within 6–12 months after the index date or patients who did not receive treatment during the selected time window.

Treatment adherence was defined as the approved dosing schedule indicated for the treatment of osteoporosis for each medication. For example, romosozumab is recommended once a month for 12 doses, denosumab once every six months, and teriparatide is administered once daily for a period of up to two years (Tu et al., 2018).

Treatment adherence was calculated as the ratio between the date of the actual purchase of AOM and the date that the medication was prescribed. The ratios were converted into a categorical variable with two levels: good adherence, defined as a ratio of 0.8 or greater, and poor adherence, defined as a ratio below 0.8.

The secondary endpoints of the study included diagnosis of osteoporosis after fragility fracture, and completion of a bone density (DEXA/DXA) scan within this overall time duration.

### 2.4 Statistical analysis

The statistical significance between patients hospitalized at the Rehabilitation Medical Center and those who did not receive rehabilitation at our medical centre was assessed using the chi-square test for categorical variables (SES and time to initiation of treatment) with post-hoc analysis in order to find the statistical significance between the categorical levels. The categories for initiation of treatment are: good, moderate and late practice and those for the SES are: high, medium and low. *t*-test was used for the continuous variables.

## 3 Results

We identified 4124 patients aged 50–85 years with fragility hip fractures between 2017 and 2021. There were no missing values during the extraction process, as per the study design. The cohort at the Rehabilitation Medical Center was younger, with a mean age of 65.47 ± 7.96 years, in comparison to other hospitals, which had a mean age of 73.06 ± 8.54 years (*p* < 0.001). The majority of patients were female, with a mean overall prevalence of 65.5 %. There was no gender distribution difference of gender between the FLS initiative and other hospitals (*p* = 0.12). The mean SES of the study cohort was categorized as medium at 63.5 %. The medium SES prevalence of the FLS initiative patients was lower than that of other hospitals (50.5 % and 63.9 % respectively), and the high SES prevalence was higher (36.9 % and 20.3 % respectively ((*p* < 0.001)). There was no difference in the prevalence of low SES between the groups (*p* = 0.21). The study cohort at the FLS initiative had higher rates of osteoporosis diagnosis (73.8 %) in comparison to other hospitals (57.6 % (p < 0.001)). There was no statistical difference between the prevalence of the bone density scans at the FLS initiative (8.1 %) and other hospitals (5.7 %, *p* = 0.28). The characteristics of the study cohort are represented in Table 1.

**Table 1:** Characteristics of study cohort.

	The FLS initiative	Other Hospitals
Cohort size, n	111	4013
Age (years), mean ± SD	65.7 ± 8.1	73.1 ± 8.5
Gender (female), n (%)	68 (59.6)	2712 (66.1)
SES^a^		
Low, n (%)	9 (8.1)	484 (12.1)
Medium, n (%)	56 (50.5)	2565 (63.9)
High, n (%)	41 (36.9)	815 (20.3)
Osteoporosis diagnosis, n (%)	82 (73.8)	2380 (57.6)
Bone density scan, n (%)	9 (8.1)	242 (5.7)

Notes; ^a^SES = socio-economic status.

Table 2 illustrates osteoporotic treatment initiation of treatment naïve patients for secondary prevention of fragility fracture. The FLS initiative showed higher rates of treatment initiation of naïve patients with 72.1 % of all patients receiving pharmacological therapy following fragility hip fracture. In comparison, 45.1 % of patients in other hospitals without FLS received osteoporosis pharmacotherapy (*p* < 0.001). The six osteoporosis medications commonly used in Israel included alendronate, risedronate, zoledronate, denosumab, and romosozumab. Table 2 lists the comparative data of the medications utilized both in the FLS initiative and other hospitals. Chi square test and post hoc analysis for each AOM initiation category versus all others were found to be statistically significant (*p* < 0.001).

**Table 2:** Osteoporotic Treatment Initiation to Treatment Naïve Patients for Secondary Prevention of Fragility Factures.

Initiated pharmacological treatment after index date (ever)	The FLS initiative (*n* = 111)	Other hospitals (*n* = 4013)	All (*n* = 4124)
Alendronate, n (%)	5 (4.5)	305 (7.6)	310
Risedronate, n (%)	6 (5.4)	366 (9.1)	372
Zoledronate, n (%)	5 (4.5)	358 (8.9)	363
Denosumab, n (%)	59 (53.1)	667 (16.6)	726
Romosozumab, n (%)	2 (1.8)	7 (0.2)	9
Teriparatide, n (%)	3 (2.7)	108 (2.7)	111
**All medications – sum,** n (%)	**80 (72.1)**	**1811 (45.1)**	**1891(45.8** **%)**

**Fig. 2:**
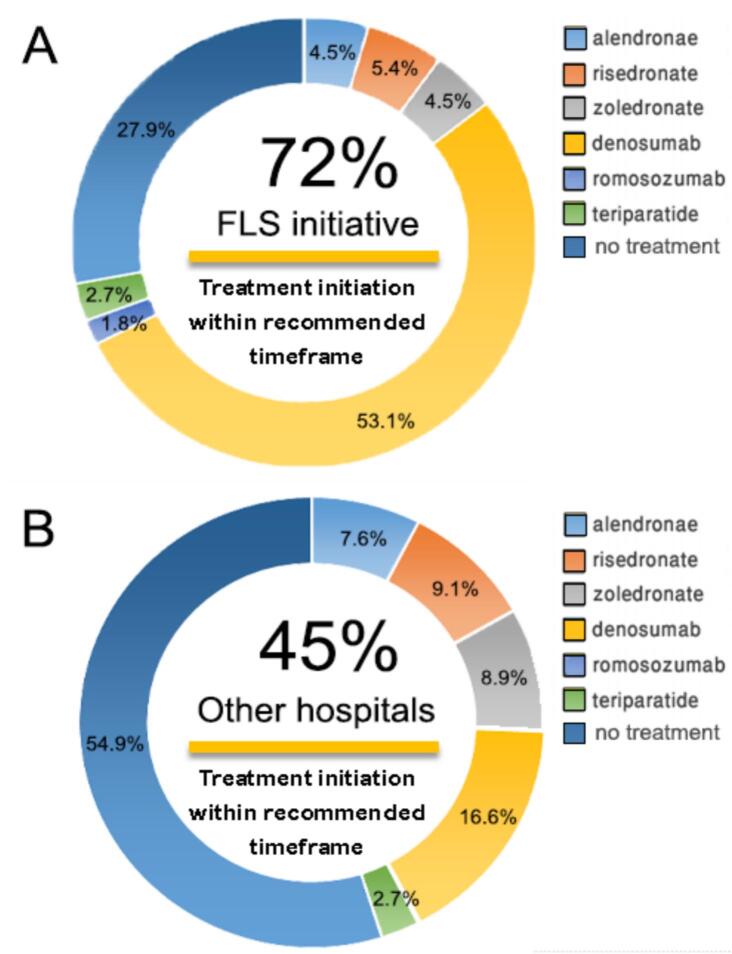
Prevalence of initiation of AOM with breakdown of treatments. 2A. Given to FLS initiative patients. 2B. other hospitals.

Fig. 2 is a pie graph that depicts time to treatment initiation for patients within the FLS initiative compared to other hospitals. 53.1 % of the FLS initiative patients received denosumab as the pharmacotherapy of choice while in other hospitals there is a larger diversity between treatment options and only 16.6 % received denosumab (*p* < 0.001). However, more patients from other hospitals received one of the oral bisphosphonate options: alendronate, risedronate or zoledronate in comparison to the FLS initiative patients 25.6 % versus 14.4 % respectively (p < 0.001).

**Fig. 3:**
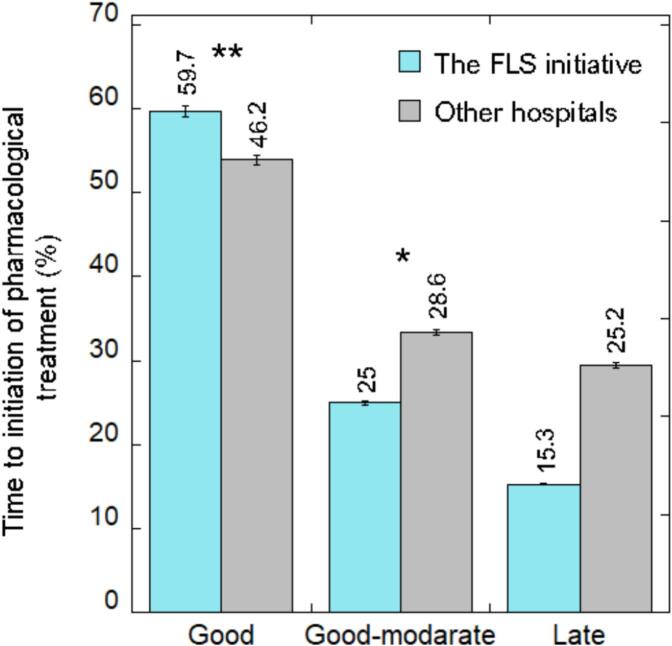
Prevalence of time to initiation of AOM. Notes;, * *p*-value<0.05, ** p-value <0.001.

Fig. 3 demonstrates the time to treatment initiation during the first year from the index date and stratifies the time according to best, good-to-moderate, and late practice. 59.7 % of patients at the FLS initiative received osteoporosis treatment within 3 months of the date of fracture, defined as best practice. In comparison, 46.2 % of patients within other hospitals received treatment during this time frame (*p* < 0.001). 25 % of patients at the FLS initiative received good-to-moderate care and were initiated osteoporosis treatment within 3 to 6 months, in comparison to 28.6 % in other hospitals (*p* < 0.05). The rate of late practice was found to be lower in the FLS initiative compared to other hospitals, 15.3 % compared to 25.2 % respectively. However, the chi-square test for late practice did not find statistical significance (*p* = 0.65).

**Fig. 4:**
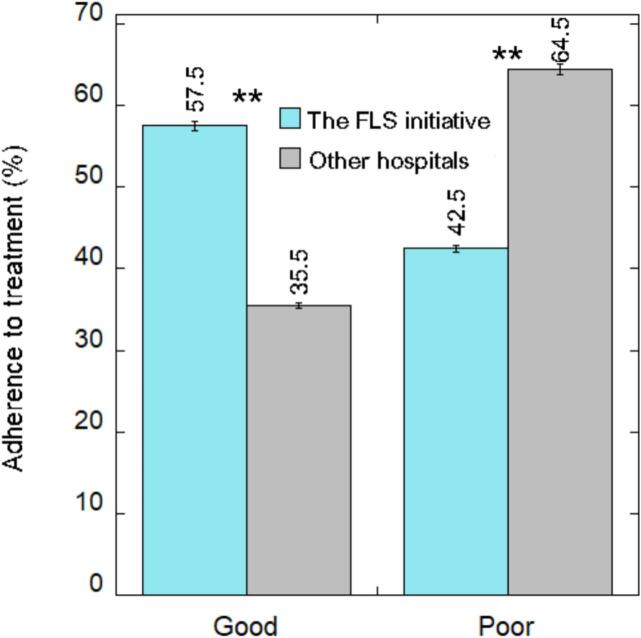
Prevalence of adherence to AOM. Note; ** p-value <0.001.

Adherence to osteoporosis treatment is illustrated in Fig. 4. Patients in the FLS initiative who initiated medical treatment, were found to have higher rates of good adherence (>0.8 ratio) compared to other hospitals, at 57.5 % compared to 35.5 % respectively (*p* < 0.001). Patients in the FLS initiative demonstrated a lower rate of poor adherence (<0.8 ratio) compared to other hospitals, with 42.5 % compared to 64.5 %, respectively. This difference was statistically significant (p < 0.001).

## 4 Discussion

This retrospective electronic medical record database study was conducted in order to assess the efficacy of the FLS initiative in the inpatient rehabilitation setting. The inpatient FLS initiative was developed in response to the well-defined care gap in osteoporosis management (Curtis et al., 2022; Greenspan et al., 2012; Svedbom et al., 2013). Initiation of pharmacological therapy for secondary prevention of fragility fractures and adherence to the course of medical therapy were selected as primary objectives to evaluate the efficacy of the FLS initiative. This study demonstrates the important role that the inpatient rehabilitation FLS initiative has in the management of osteoporosis. The study found higher rates of osteoporosis diagnosis, and higher rates of AOM initiation within the recommended timeframe of three months, defined as good practice, and within 3 to 6 months, defined as good-moderate practice. The FLS initiative also demonstrated higher adherence rates to prescribed AOM compared to other hospitals.

According to the latest report of the National Program for Quality Indicators in community healthcare in Israel, the rate of AOM treatment during the first year following hip fracture has increased slightly, from 25.5 % in 2015 to 28.1 % in 2018, with a goal of reaching at least 50 % treatment (Bernstein et al., 2022). This study demonstrated higher rates of treatment initiation for patients requiring secondary fracture prevention. The FLS initiative demonstrated an AOM treatment initiation rate of 72 % among treatment naïve patients compared to 45 % in other hospitals during 2017 to 2021. The results of this study emphasise the need for establishing FLS initiatives at rehabilitation centres as a means to identify, treat, and monitor patients with osteoporosis in order to address the care gap effectively.

The prescription rate of osteoporosis medications might help distinguish between a general lack of focus on osteoporosis care and a specific reluctance to prescribe these treatments. Although we do not have data on the prescription rates of calcium or vitamin D in our dataset, the National Program for Quality Indicators in Hospitals recommended routine vitamin D administration at discharge following hip fractures. According to Ministry of Health reports, compliance with this recommendation has been high (94 %–97 %) (Quality and Safety Division Health Services Research Department, 2021). These data suggest that post-fracture care is indeed prioritized, and that the gap lies not in overall management, but specifically in the underuse of AOM.

The Rehabilitation Working Group of the IOF Committee of Scientific Advisors characterized the range of rehabilitation modalities instrumental in the management of fragility fractures. The review emphasised the importance of rehabilitation following a fragility fracture. However, they recommended post-fragility interventions such as pain reduction, physiotherapy, patient education, and nutrition (Pinto et al., 2022). Only a few studies focused on the implementation of the FLS model at rehabilitation centers (Lebanon et al., 2020; Cosman et al., 2017). Cousman et al. demonstrated how the rehabilitation FLS initiative improved non-pharmacological management (vitamin D and calcium intake) but not AOM use (Cosman et al., 2017).

Lebanon et al. illustrated the positive effects of virtual FLS intervention that incorporated a combined orthopaedic, rehabilitation, and metabolic team approach on patients with fragility hip fractures (Lebanon et al., 2020). However, the postintervention OAM issue rate remained low, below 50 % in their treatment group. In comparison, our FLS initiative showed significant higher rates of treatment initiation of 74 %. Our study shows that treating patients at an inpatient rehabilitation FLS that incorporates identification, treatment, comprehensive management, monitoring, and follow-up following fragility hip fracture dramatically increases the rate of AOM treatment and effectively addresses the care gap.

The majority of the patients at the FLS initiative were treated with denosumab, while other hospitals demonstrated lower prescribing rates of 53.1 % and 16.6 % respectively. The cohort of patients hospitalized at the rehabilitation hospital who were included in the FLS initiative was all categorized as very-high risk for secondary fractures, due to being hospitalized for a fracture in the previous 12 months (Kanis et al., 2020). Very-high-risk patients require potent therapy for secondary fracture prevention. Until 2020, denosumab was the most potent first-line pharmacotherapy for post hip fracture patients available through the hospitals. In that year, other agents became available as first line treatments for these patients, including teriparatide and romosozumab. Our study demonstrates that post hip fracture patients identified and treated through the FLS initiative received a treatment protocol that more closely corresponded to the international guidelines for the treatment of osteoporosis that called for more potent therapies for very high-risk patients.

Several factors explain the higher prescribing rates of denosumab compared to other treatments. Denosumab was found to be more effective than bisphosphonates in improving bone density (Deeks, 2018). It is also widely recognised that adherence to oral bisphosphonates is poor (Fatoye et al., 2019), and adherence to denosumab is higher than treatment with bisphosphonates (Li et al., 2015). A large retrospective, observational cohort study followed 10,863 female patients in the US who were newly initiated on AOM. The study assessed persistence and compliance following initiation of osteoporosis medications, including denosumab, alendronate, ibandronate, risedronate, raloxifene, and teriparatide. The rates of persistence and compliance over 12 months were higher among women initiated with denosumab compared with those initiated with other osteoporosis therapies. Bisphosphonates were associated with the lowest rates of compliance (Li et al., 2015). Therefore, denosumab was preferred in our FLS initiative to improve adherence rates and treatment outcomes.

Other hospitals without an FLS initiative may have concerns with prescribing denosumab due to the risk of rebound fractures with missed or delayed doses. This problem is less of a concern in the rehabilitation department setting, as longer hospitalizations allow for comprehensive patient education regarding the use and importance of adhering to the drug of choice. Cognitive assessments of patients, including the Mini-Mental State Exam (MMSE) and Montreal Cognitive Assessment (MOCA) exams, are routinely completed for every rehabilitation inpatient, thereby reassuring the FLS staff of the patient's cognitive ability to follow the treatment protocol carefully.

The proportion of patients who underwent bone density testing was 8.1 % in the FLS initiative and 5.7 % in other hospitals. This difference was not statistically significant (*p* = 0.28).

In U.S.-based studies, the reported rate of DXA testing after hip fracture ranges from 4 % to 12 % (Rudisill et al., 2024; Galli et al., 2022; Ross et al., 2021). Among patients aged 65–69, higher rates of 10 % to 23 % have been reported (Rudisill et al., 2024; Ross et al., 2021). Given that the mean age in this study was 65–75, a higher rate of DXA testing in the primary care setting might have been expected. However, it is important to note that, according to international guidelines, a DXA scan is not required to initiate treatment for secondary prevention after a fragility hip fracture (Osteoporosis overview, 2020). The occurrence of a low-energy hip fracture alone is sufficient to establish a diagnosis of osteoporosis and justify pharmacologic treatment. In this context, the primary purpose of a baseline DXA scan is to support monitoring of treatment response over time, rather than to guide the decision to start therapy.

A study from Israel (Soroka University Medical Center) reported that before the implementation of a fracture liaison service (FLS), the rate of DXA testing after hip fracture was 7.5 %. Following the introduction of FLS, this rate increased to 34 % (Yoel et al., 2025).

Therefore, the relatively low rate of DXA testing in this study, particularly within the first year after fracture in the FLS rehabilitation center, may reflect an appropriate focus on the timely initiation of treatment without unnecessary delays. Additionally, the relatively short follow-up period may not have provided sufficient time for DXA testing to be performed for monitoring purposes.

The robust FLS at the rehabilitation hospital incorporated working methods to promote patient adherence. These interventions included patient education, in-house follow-up, and call-back services by the FLS co-ordinator every six months. This intensive follow-up ensured that patients were more likely to receive subsequent denosumab doses as scheduled. Since the risk of rebound fractures was minimized, FLS physicians felt more confident prescribing denosumab to patients. In order to promote accurate follow-up, in December 2021, the rehabilitation hospital inaugurated the FLS outpatient clinic. The clinic allowed for the expansion of services, including laboratory, radiology, and multi-disciplinary investigations, to include discharged FLS patients during routine ambulatory follow-up visits.

## 5 Limitations

This research was conducted on an anonymized electronic medical records database. The use of data extraction from a broad population provides a more efficient method for analyzing large datasets (Willis et al., 2019). However, anonymization prevented direct inquiry into the reasons for poor adherence or delayed initiation of AOM treatment. Additionally, in the absence of patient interviews, data on side effects contributing to low adherence were not available.

Information on geographical factors such as the physical and built environment (transportation, subways, bus stops, etc.) may also impact the initiation and adherence to treatment, and needs to be further investigated in a geographical design study. Spatial analysis that utilizes geographic data and includes other sociodemographic factors, such as caregiver support and education level, may reveal a novel perspective on secondary prevention of fragility fractures and osteoporosis treatment.

The FLS initiative group is younger, with an average age of 65 compared to 73 in other hospitals that represent the general population. However, the majority of patients with fragility hip fractures are female, consistent with findings in the literature (Camacho et al., 2020). Additionally, the cohort primarily includes patients from medium and high SES groups, with lower SES populations being underrepresented. Further research is needed to examine the generalisability of the results to lower SES groups, as they may differ in the initiation and adherence to AOM treatments due to varying circumstances.

Another limitation of this study is the difference in length of stay between patients in the rehabilitation setting and those in general hospitals, typically in orthopaedic departments. Although specific length-of-stay data were not collected, rehabilitation admissions in this context are generally longer than stays in acute hospitals (Hommel et al., 2008). A longer hospitalization may offer more opportunity to assess patients and initiate secondary fracture prevention. However, without a dedicated FLS program, these opportunities may not lead to a reduction in the care gap, as medical teams are not systematically focused on osteoporosis management. This suggests that the structured approach of the FLS, rather than length of stay alone, played a key role in improving treatment initiation and adherence.

Additionally, data from a single center can potentially have a disproportionate influence on the overall results. However, we believe this is unlikely for several reasons. All non-FLS hospitals included in this study participate in the National Program for Quality Indicators in Hospitals, which ensures a standardized approach to key aspects of fragility fracture care, including timely surgery (within 48 h) and prescription of vitamin D at discharge during the study period (Quality and Safety Division Health Services Research Department, 2021). National health reports indicate high and uniform adherence to these quality indicators across all centers, regardless of hospital size or specific characteristics of their orthopaedic departments. While minor differences may exist between hospitals, the overall model of care for fragility fractures and secondary prevention of osteoporosis is well established across the region. Therefore, we consider it unlikely that an outlier hospital drove the low rate of AOM prescription observed in the non-FLS group. Instead, these findings likely reflect a broader, systemic care gap in secondary prevention after fragility fractures.

## 6 Conclusions

Secondary prevention of fragility hip fracture with FLS initiative in the inpatient rehabilitation setting was found to be highly effective in improving time to treatment initiation and adherence to AOM. The rehabilitation FLS model is an effective model that can address the care gap, improve patient outcomes and decrease osteoporosis related morbidity and mortality. In this model, denosumab was most often prescribed and therefore may partially explain the positive adherence rate.

## CRediT authorship contribution statement

**Orit Mazza:** Writing – review & editing, Writing – original draft, Visualization, Validation, Methodology, Investigation, Data curation, Conceptualization. **Chemda Gluck:** Writing – review & editing, Writing – original draft, Project administration. **Noa Menkes-Caspi:** Formal analysis, Data curation. **Robyn Jacob Bornstein:** Writing – review & editing, Writing – original draft, Conceptualization. **Hagay Amir:** Writing – review & editing. **Michael Bahar:** Writing – review & editing, Writing – original draft, Methodology, Conceptualization. **Amir Haim:** Writing – review & editing, Writing – original draft, Project administration, Methodology, Conceptualization.

## Declaration of Generative AI and AI-assisted technologies in the writing process

During the preparation of this work the authors used ChatGPT4o in order to improve readability and language in small parts of the manuscript. After using this tool, the authors reviewed and edited the content.

## Funding

This research did not receive any specific grant from funding agencies in the public, commercial, or not-for-profit sectors.

## Declaration of competing interest

The authors declare that the research was conducted in the absence of any commercial or financial relationships that could be construed as a potential conflict of interest.

## Data Availability

The authors do not have permission to share data.
